# Indium tin oxide exhibiting high poly-crystallinity on oxygen plasma-treated polyethylene terephthalate surface

**DOI:** 10.1186/1556-276X-7-118

**Published:** 2012-02-13

**Authors:** Phil Kook Son, Suk-Won Choi, Sung Soo Kim

**Affiliations:** 1 Department of Advanced Materials Engineering for Information & Electronics, Kyung Hee University, Yongin, Gyeonggi-do, 446-701, Republic of Korea; 2 Department of Chemical Engineering, Kyung Hee University, Yongin, Gyeonggi-do, 446-701, Republic of Korea; 3 Regional Innovation Center-Components and Materials for Information Display, Kyung Hee University, Yongin, Gyeonggi-do, 446-701, Republic of Korea

**Keywords:** ITO film, XRD, poly-crystal, PET, low resistivity

## Abstract

Low-resistivity indium tin oxide [ITO] film was successfully deposited on oxygen plasma-treated polyethylene terephthalate [PET] surfaces at room temperature. X-ray diffraction [XRD] measurements demonstrated that the film deposited on the PET surface that had not been treated with oxygen plasma had an amorphous structure. In contrast, after the low-power oxygen plasma treatment of the PET surface, the ITO film deposited on the PET surface had a poly-crystalline structure due to interactions between electric dipoles on the PET surface and electric dipoles in the ITO film. The minimum resistivity of the poly-crystalline ITO was about 3.6 times lower than that of the amorphous ITO film. In addition, we found that the resistivity of ITO film is proportional to the intensity of the (400) line in the film's XRD spectra.

## Background

Transparent conducting oxide [TCO] films have been widely used as transparent electrodes in the field of optoelectronic devices. Specifically, they have been used in applications such as liquid crystal displays, organic light-emitting diodes, e-papers, and thin-film solar cells [1-4]. It is well known that the performance of such devices is closely related to the physical properties (for example, the electrical and optical properties) of the TCO [5]. Indium tin oxide [ITO] is the most widely used TCO because of its excellent electrical and optical characteristics. However, ITO requires high process temperatures (greater than 200°C) in order to obtain these favorable characteristics. This is because an additional annealing treatment is needed to transform its structure from amorphous to poly-crystalline. Thus, it is difficult to apply ITO films onto flexible plastic substrates such as polyethylene terephthalate [PET], polyimide, polyethylene naphthalate, and polycarbonate because these polymer substrates have low glass transition temperatures [6]. In general, there are several disadvantages to fabricating ITO on plastic substrates as opposed to glass substrates, which can be fabricated with a relatively low treatment temperature. These disadvantages include low transmittance, low conductivity, and many defects. Therefore, the development of a low temperature process for depositing ITO onto plastic substrates is very important.

In this work, we present an intriguing technique for fabricating high-performance ITO film on PET without an additional annealing process. Using this method, ITO film with a poly-crystalline structure can be manufactured on oxygen plasma-treated PET surfaces without an additional annealing process. The physical properties of the deposited ITOs on the oxygen plasma-treated PET surfaces are discussed as a function of oxygen plasma power. It is found that the poly-crystalline content of ITO can be optimized by controlling the oxygen plasma power on PET surface.

## Methods

ITO films were deposited onto non-treated PET using a radio frequency magnetron sputtering system and low-power oxygen plasma-treated PET. Argon (49.7 sccm) was used as the inert gas in the chamber, and oxygen (0.3 sccm) was also used in the chamber. The base pressure of the deposition chamber was around 10^-6 ^Torr, and the working process pressure was around 10^-2 ^Torr. ITO films with a thickness of 50 nm were deposited at room temperature. The oxygen plasma-treated PET surfaces were bombarded by a low-power oxygen gas. The oxygen plasma power, incident angle, and exposure time were 30 to 100 W, 70°, and 5 s, respectively.

X-ray diffraction [XRD] measurements of the SiOx film were performed using an X-ray diffractometer (X'Pert Pro, Philips, PANalytical B.V., Almelo, The Netherlands) equipped with monochromic CuK*α *radiation (*λ *= 1.054056 Å) operated at 40 kV and 30 mA. The diffraction pattern was measured at room temperature in normal *θ*-2*θ *scanning mode over angles ranging from 10° to 90° with a step of 0.05°, and measurements were performed at a rate of 0.2 s/step.

We also characterized the film's surface morphology using atomic force microscopy [AFM] in the tapping mode (Multimode AFM Nanoscope IIIa, Digital Instruments, Inc., Tonowanda, NY, USA). An ultra-lever cantilever with a spring constant of 26 N/m and a resonance frequency of 268 kHz was used for scanning. Optical transmittance measurements were carried out with an UV-Vis NIR spectrometer.

The wetting properties of the surfaces were determined by the static contact angle method. The contact angles were measured by increasing and then decreasing the volume of a drop of liquid (distilled water) deposited on the sample surface. Recorded images were digitized and analyzed with a software routine that evaluated the tangent at the point of contact between the drop and the surface (i.e., the contact angle).

Measurements to determine the resistivity of the thin films were performed using a four-point probe test. Rectangular sections (3 × 1 cm^2^) were cut from the substrate. The surfaces of the sections were cleaned thoroughly before the resistivity measurements were made. The four-point probe was placed in contact with the surface of the film, and a fixed current of 10 mA was applied across the outer two probes. The voltage drop across the two inner probes was measured.

## Results and discussion

**Figure 1:**
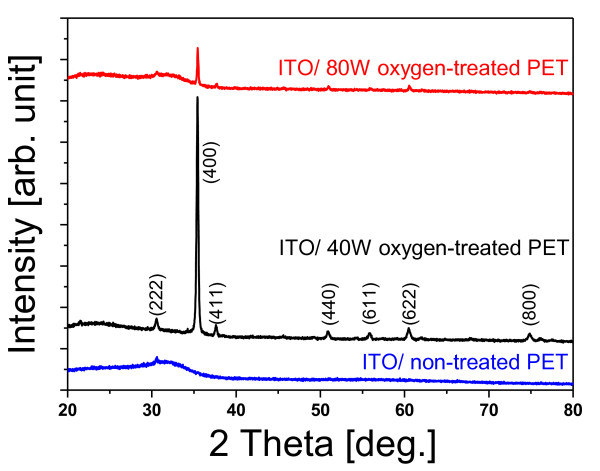
**XRD spectra for ITO films deposited on PET films**. These ITO films were deposited on non-treated and oxygen plasma-treated (40 and 80 W, respectively) PET films without any additional annealing process.

Figure 1 shows the XRD results for ITO films deposited on non-treated and oxygen plasma-treated PET film without any additional annealing process. From the XRD data, it was found that amorphous ITO was deposited on the non-treated PET, while poly-crystalline ITO was deposited on the oxygen plasma-treated PET. Specifically, poly-crystalline ITO was formed on the PET treated with an oxygen plasma power of 40 W. In the cases where oxygen plasma treatment was performed with a power greater than 50 W, from the intensity of the (400) line in the XRD spectra, it was found that the proportion of poly-crystalline structure began to decrease. Therefore, it was found that there was an optimal condition for the oxygen plasma treatment of PET to maximize the poly-crystalline content of the deposited films.

**Figure 2:**
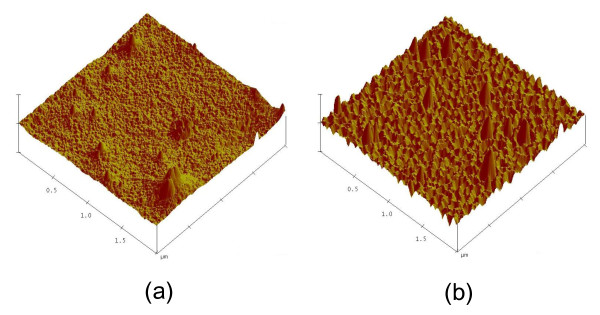
**AFM images of (a) non-treated and (b) oxygen plasma-treated PET surfaces**.

**Figure 3:**
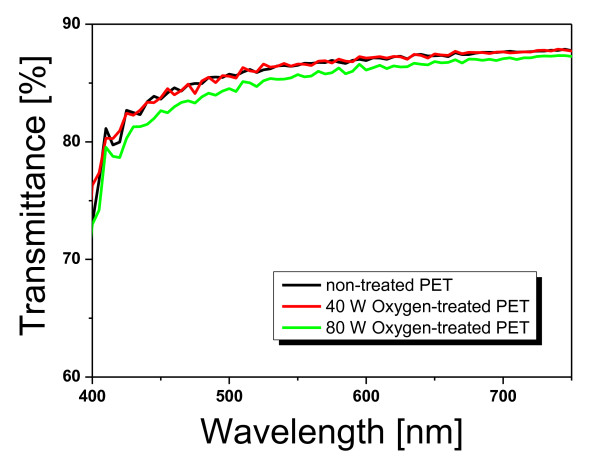
**Transmittance curves of non-treated and oxygen plasma-treated (50 and 80 W, respectively) PET films**.

To obtain more information about the oxygen plasma-treated PET surfaces, we measured the morphologies and transmittance properties of the PET surfaces. Figure 2a, b shows the morphologies of non-treated and oxygen plasma-treated PET surfaces, respectively, measured via AFM. The observed surface roughness was increased by the oxygen plasma treatment. The surface roughness remained constant at approximately 2.85 ± 0.6 nm irrespective of the oxygen plasma power (approximately 100 W). As shown in Figure 3, the transmittance of the PET film treated below the optimum oxygen plasma power of 40 W was similar to that of non-treated PET film. In contrast, the transmittance of PET film treated at oxygen plasma powers above 50 W was slightly lower than that of the non-treated PET film.

**Figure 4:**
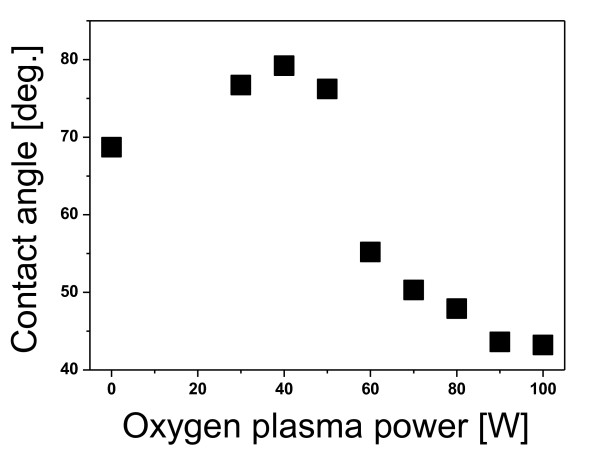
**Measured contact angles on oxygen plasma-treated PET surfaces as a function of oxygen power**.

**Figure 5:**
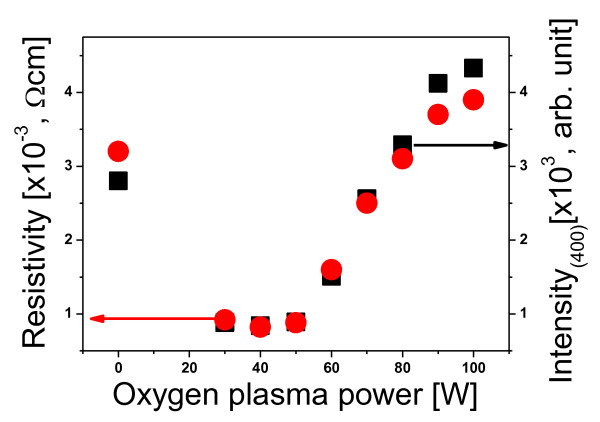
**Film evaluated resistivity and (400) line of XRD intensity as a function of oxygen power**.

Figure 4 shows the measured contact angles on PET surfaces as a function of the oxygen plasma power. As shown in Figure 4, the contact angle was altered dramatically by the oxygen plasma power. In the range of approximately 30 to 50 W, the deposited ITO exhibited a poly-crystalline structure, and the evaluated contact angle was relatively high (about 76° to 80°). When the power was greater than 50 W, the poly-crystallinity of the deposited ITO was decreased, and the contact angle was below 55°. From the contact angle experimental data, we found that the oxygen plasma-treated PET film had a high contact angle. This is due to the surface charges (electric dipole moments) on the PET film surface [7]. We suggest that electric dipole-dipole interactions between the ITO film and the oxygen plasma-treated PET surfaces play a dominant role in the formation of poly-crystalline ITO on low-energy ion beam-treated inorganic surfaces. Figure 5 shows the resistivity of the ITO on PET as a function of the oxygen plasma power. The observed resistivity of the ITO deposited onto a surface treated with 40 W of oxygen plasma was considerably lower than that of the ITO deposited on non-treated PET. However, the observed resistivity of the deposited ITO increased from 0.9 × 10^-3 ^Ω cm to 3.9 × 10^-3 ^Ω cm as the oxygen plasma power was increased from 50 to 100 W. We also measured the intensity of the (400) line of XRD as a function of the oxygen plasma power. As shown in Figure 5, the curve of the resistivity is similar to that of the intensity of the (400) line of XRD. The similarity of these trends indicates that the electrical property strongly depends on the poly-crystallinity of the deposited ITO. From our measurements, the maximum electrical conductivity of the ITO on the oxygen plasma-treated PET was approximately 3.6 times higher than that of the ITO on non-treated PET.

## Conclusion

We successfully manufactured ITO that exhibits high poly-crystalline content on oxygen plasma-treated PET surfaces without an additional annealing process. Before the oxygen plasma treatment of the PET surface, the ITO deposited on the PET surfaces exhibited an amorphous structure. In contrast, after the oxygen plasma treatment on PET film, the ITO deposited on the PET surfaces had a poly-crystalline content that depended on the oxygen plasma power. We found that the optimum poly-crystallinity of ITO could be achieved by controlling the oxygen plasma power used on the PET surface. The minimum resistivity of the ITO on the oxygen plasma-treated PET was about 3.6 times lower than that of the ITO on non-treated PET.

## Competing interests

The authors declare that they have no competing interests.

## Authors' contributions

PKS and SWC acquired and interpreted the data, carried out the analysis, and drafted the manuscript. PKS participated in XRD and AFM measurements. PKS acquired the optical transmittance, contact angle, and resistivity data. SWC and SSK have been involved in revising the manuscript and have given the final approval of the version to be published. All authors read and approved the final manuscript.
